# Management of patient with Sturge-Weber syndrome: a case report

**DOI:** 10.1186/1757-1626-2-9394

**Published:** 2009-12-23

**Authors:** Valbona Govori, Bujar Gjikolli, Halil Ajvazi, Nada Morina

**Affiliations:** 1 Neurology Clinic, University Clinical Center of Kosova, Prishtina, Republic of Kosova; 2 Institute of Radiology, University Clinical Center of Kosova, Prishtina, Republic of Kosova; 3 Clinic of Ophthalmology, University Clinical Center of Kosova, Prishtina, Republic of Kosova; 4 Psychiatry Clinic, University Clinical Center of Kosova, Prishtina, Republic of Kosova

## Abstract

**Introduction:**

Sturge-Weber syndrome sometimes referred to as encephalotrigeminal angiomatosis, is a rare congenital neurological and skin disorder.

**Case presentation:**

This is case report of a 18-year-old mentally disabled boy, with long-standing seizures, with a port-wine nevi on the left side of the face along the distribution of trigeminal nerve. Interictal encephalogram showed bilateral slow activity, pronounced in the left hemisphere, with epileptogenic activity in the left temporo-parietal region. Skull radiograph, computerized tomography and magnetic resonance imaging showed intracranial calcifications and atrophy of the left brain hemisphere.

**Conclusion:**

Professional counseling and support in addition to drug treatment can provide help to patients and their family to overcome their problems and improve the treatment outcome.

## Introduction

The Sturge-Weber syndrome is a neurocutaneus congenital but not an inherited disease and it occurs sporadically. It is a disorder of vasculature which belongs to the group of phacomatoses characterized by nevus flammeus and angiomas of the meninges [1]. It is a rare disease characterized by a birth mark called port wine nevi, associated with abnormalities of the brain caused by abnormal blood vessels (angiomas) that occur on the cerebral cortex [2,3]. These changes are usually unilateral. It can be seen in both sexes equally, and no racial differences have been identified. Port wine nevi are congenital malformations in the dermis of the skin involving venules, capillaries and possibly perivenular nerves [4].

## Case presentation

An 18 years old mentally disabled boy living in a rural part of Kosova developed seizures at the age of 4 months. At that time he was hospitalized and treated in the Department of Neurology in Skopje-Macedonia. The diagnosis, Sturge Weber syndrome, given to him during that time was based only on clinical signs: port-wine nevi on the left side of the face along the distribution of the trigeminal nerve, generalized seizures, and hemiparesis of the right side of the body. Additionally, the patient has received Phenobarbital 30 mg per day and used the medication for some months. Because of poor financial conditions, the patient's family could not provide him regular medication. From that time, the patient had regular partial seizures (right-sided tonic clonic seizures with facial twitch) that lasted 2-3 minutes with a frequency of 2-3 times per day. While he was experiencing these frequent seizures (for years) the only treatment he received was some cold wet towels placed on his forehead.

Thus he had not been treated until 2005 when his family brought him for the first time in our Clinic of Neurology in Prishtina. Patient was brought in the Clinic of Neurology with severe complex partial seizures (no EEG recording is shown in presentation), presenting with a history of epilepsy which was not well controlled.

**Figure 1:**
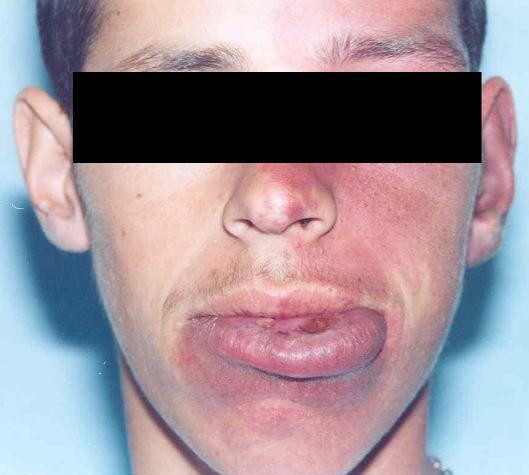
**Port-wine stain (front and side views) in the left half of the face, neck and lips**.

**Figure 2:**
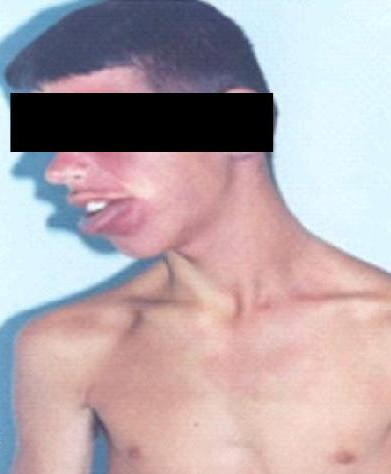
**Port-wine stain (front and side views) in the left half of the face, neck and lips**.

Physical examination revealed port wine nevi, localized in the complete left half of the face, including left half of the neck (Figure 1 and Figure 2) with right sided hemi paresis. Phenobarbital was excluded by his mother, thus we started treatment with carbamazepine 600 mg per day. In addition to the treatment of the patient, efforts were made to educate the family members about the regular treatment of the patient. Since then, patient has been seizure free for almost five years.

**Figure 3:**
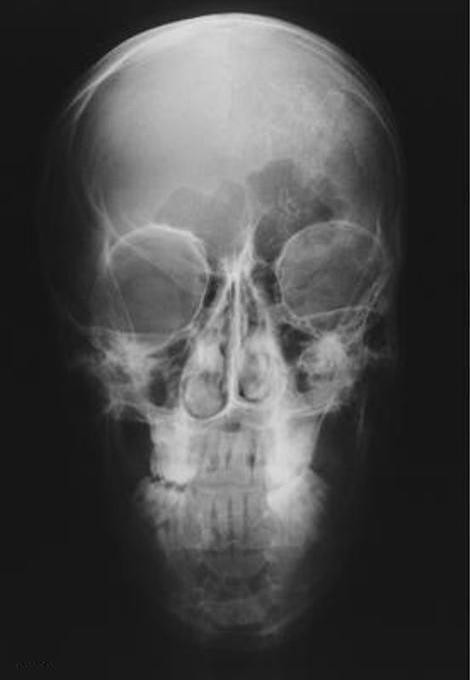
**X ray of the skull in standard projections reveals intracranial calcifications in the form of "tram lines" in the left hemisphere of the brain**.

**Figure 4:**
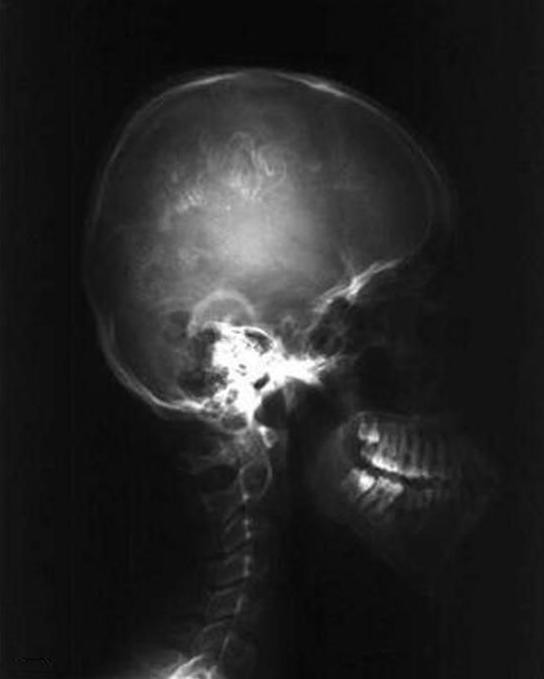
**X ray of the skull in standard projections reveals intracranial calcifications in the form of "tram lines" in the left hemisphere of the brain**.

X ray of the skull showed confluent "tram-line" calcifications, from the projection of the left frontal sinus towards the posterior part of the left parietal region (Figure 3 and Figure 4).

**Figure 5:**
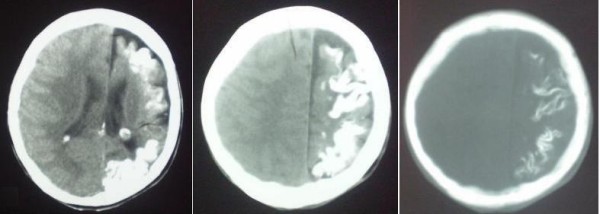
**Cranial Computed Tomography shows cortical and sub cortical gyriform calcifications of the left brain hemisphere and cortical atrophy**.

In addition, computerized tomography has been performed and gyriphorm calcifications with atrophy of left hemisphere have been shown (Figure 5).

**Figure 6:**
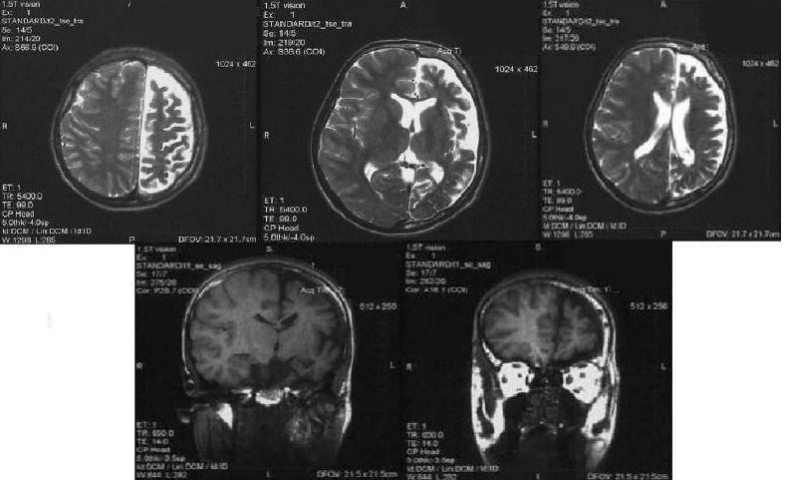
**Magnetic resonance imaging of the brain, axial T2 and coronal T1 reveal atrophy of left hemisphere with signal void in sub cortical areas and wider left ventricle compared to the right one**.

MRI of the brain was performed and it revealed severe left cerebral hemi atrophy. Left ventricle was wider compared to the right ventricle (Figure 6).

Inter-ictal encephalogram showed bilateral slow wave activity, greater over the left side, with epileptogenic activity in the left temporo-parietal region.

The patient was referred for neuropsychological and neuro-ophthalmologic examination.

Psychological examination revealed IQ = 52 (Goodinaph test, Kohs test). He also showed latent aggressive tendencies and emotional imbalance.

**Figure 7:**
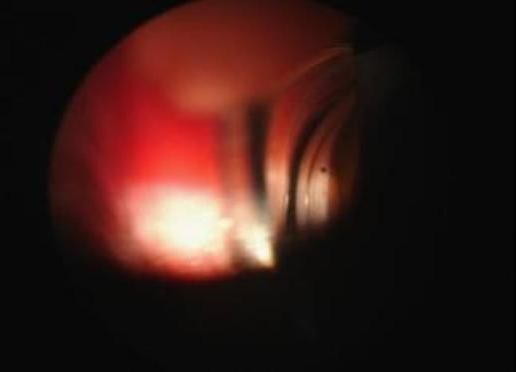
**Ophthalmologic examination shows congestion of blood vessels**.

**Figure 8:**
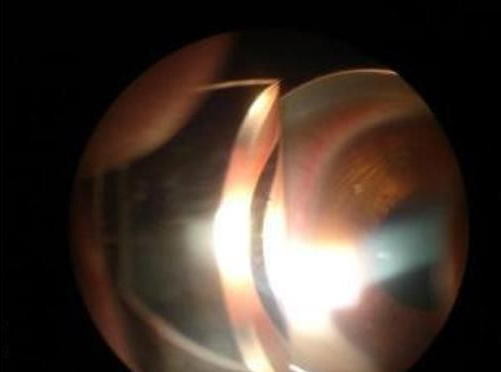
**Ophthalmologic examination shows congestion of blood vessels**.

Ophthalmologic examination of the left eye showed congestion of blood vessels (Figure 7 and Figure 8), initial compensated glaucoma with increased ocular tension of 24 mmHg, papilla nerve optic had excavation of 0.3-0.4, deflection D = I. Irido corneal angle was opened and presented neovascularisation and Schlem's channel was filled with blood. The right eye was unremarkable.

## Discussion

Sturge-Weber Syndrome is a congenital but not inherited disease. It is neurocutaneous syndrome presented with vascular malformations resulting from the failure of fetal veins to develop normally, changes in the brain, skin, and eye. These malformations lead to venous hypertension and subsequent hypoperfusion of the underlying cortex causing chronic cerebral ischemia, atrophy, and neurological deterioration. Sturge-Weber syndrome is a rare disease in the group of phakomatoses that cause physical, psychological, and social disorders. This syndrome occurs with equal frequency in both sexes, with seizures typically developing in the first year of life [5,6].

This is a case report of a young patient who has type one of Sturge-Weber Syndrome according to the Roach Scale classification. It consists of cerebral calcifications, birth mark, seizures, glaucoma, hemi paresis, mental retardation, and cerebral atrophy [7-9]

Neurological deficit is caused by the intracranial vessels malformation [10]. Imaging findings consist of cortical calcifications - tram line calcifications, cortical atrophy, enlarged ipsilateral choroid plexus, pial angiomatosis [11]. The best imaging modality is MRI while calcifications can be assessed in detail on CT images. The early onset of seizures prior to the age of 2 years is related to a poor prognosis with mental retardation, refractory epilepsy [12], because of the larger involvement of brain dysfunction.

Ophthalmologic abnormality is common in cases when port wine nevi are distributed in the ophthalmic and maxillary division of trigeminal nerve.

Most cases with Sturge-Weber syndrome are not life threatening. This is a progressive disease, associated with continuous neurological decline [13]. With vigorous control and treatment of symptoms, such as seizures, visual problems, paralysis and mental disorder, quality of life can be preserved.

A detailed history, physical and mental state examination, neuropsychological, neuroimaging and laboratory investigations were undertaken in our case. Our patient developed seizures at the age of 4 months. Sturge Weber syndrome has been reported in neonates as well - a case of 2 days old baby [14] and seizures are seen in about 75 to 90% of patients with Sturge-Weber syndrome [15].

Our patient had port - wine nevi on the left side of the face along the distribution of all three branches of the trigeminal nerve. Facial capillary vascular malformation-port-wine stain is common in the pediatric population, a study of 106 patients with port-wine stains, Enjolras et al concluded that patients with lesions located in the ophthalmic and trigeminal distribution areas are at risk for associated neuro-ocular symptoms [16].

In our patient, ophthalmologic examination of the left eye showed congestion of blood vessels, initial compensated glaucoma with increased ocular tension of 24 mmHg. The right eye was unremarkable. Glaucoma can be seen in up to 70% of cases with Sturge - Weber syndrome [15].

X ray of the skull showed confluent "tram-line" calcifications, while computerized tomography has shown the gyriphorm calcifications with atrophy of left hemisphere confirmed also with MRI associated with mental retardation our patient has. Cortical calcifications present at birth are reported in 30% [17] In cases of development delay and mental retardation, 50 to 60% of patients with Sturge - Weber syndrome are affected [15].

In this case, seizures were controlled with Carbamazepine 600 mg.

## Conclusion

In this case report we emphasize that regular treatment of the patient with Sturge-Weber with carbamazepine 600 mg results in long term seizure free. A successful early treatment results in control of seizures and prevention of complications.

Additionally, we strongly emphasize that professional counseling and support in addition to drug treatment can provide proper assistance to patients and their family to overcome their problems and improve the outcome of the treatment.

## Consent

Written informed consent was obtained from the patient for publication of this case report and accompanying copy of the written is available for review by the Editor-in-Chief of this journal.

## Competing interests

The authors declare that they have no competing interests.

## Authors' contributions

VG performed the examination of the patient, collected the data, and analyzed them. VG has treated the patient and performed the follow up. BGJ has analyzed the images, and assist in writing the text. HJ performed the eye examination and treatment. NM performed neuropsycological examination. All authors read and approved the final manuscript.

## References

[B1] Griffiths PD Sturge Weber Syndrome revisited: the role of neuroradiology Neuropediatrics 1996 27 284 294 10.1055/s-2007-973796 9050045

[B2] Thomas-Sohl KA Vaslov DF Maria BL Sturge-Weber syndrome: a review Pediatr Neurol 2004 30 303 10 10.1016/j.pediatrneurol.2003.12.015 15165630

[B3] Castilo M Neuroradiology companion 2006 Lippincott W&W, Philadelphia 231 233

[B4] Barsky SH Rosen S Geer DE Noe JM The nature and evaluation of port wine stains: A computer assisted study J Invest Darmatol 1980 74 154 157 10.1111/1523-1747.ep12535052 7359006

[B5] Bodensteiner JB Roach ES Bodensteiner JB, Roach ES Sturge-Weber syndrome: Introduction and overview Sturge-Weber Syndrome 1999 Sturge-Weber Foundation, Mt. Freedom, NJ 1 10

[B6] Paller AS The Sturge-Weber syndrome Pediatr Dermatol 1987 4 300 304 10.1111/j.1525-1470.1987.tb00797.x 3328186

[B7] Takeoka M Riviello JJ Sturge Weber Syndrome http://emedicine.medscape.com/article/1177523-overview

[B8] Comi AM Pathophysiology of Sturge-Weber syndrome J Child Neurol 2003 18 509 516 10.1177/08830738030180080701 13677575

[B9] Chapieski L Friedman A Lachar D Psychological functioning in children and adolescents with Sturge-Weber syndrome J Child Neurol 2000 15 660 665 10.1177/088307380001501004 11063079

[B10] Sturge WA A case of partialepilepsy apparently due to a lesion of one of the vasomotor centres of the brain Trans Clin Soc Lond 1879 12 162 167

[B11] Osborn A Salzman K Diagnostic imaging: Brain 2004 Salt Lake City: Amyrsis I-1-94-97

[B12] Sujanski E Conradi S Outcome of Sturge-weber syndrome in 52 adults Am J Med Gen 1995 57 1 35 45 10.1002/ajmg.1320570110 7645596

[B13] Rochkind S Hoffman HJ Hendrick EB Sturge Weber Syndrome: natural history and prognosis J Epilep 1990 3 293

[B14] Zhuo BY Lu GJ Ye ZZ A case of Sturge Weber syndrome Zhonghua Er Ke Zq Zhi 2004 42 944 15733372

[B15] Kugler RM Sturge - Weber syndrome affects skin and brain http://rarediseases.about.com/od/rarediseasess/a/sturgeweber.htm

[B16] Enjolras O Riche MC Merland JJ Facial port wine stains and Sturge - Weber syndrome Pediatrics 1985 76 1 48 51 4011357

[B17] Khan NA Tunbull I MacDonald S Sturge - Weber syndrome http://emedicine.medscape.com/article/414222-overview

